# Evaluation of Long Sea Snail *Hinia reticulata* (Gastropod) from the Middle Adriatic Sea as a Possible Alternative for Human Consumption

**DOI:** 10.3390/foods9070905

**Published:** 2020-07-09

**Authors:** Alberto Felici, Nina Bilandžić, Gian Enrico Magi, Nicolaia Iaffaldano, Elisa Fiordelmondo, Gerardo Doti, Alessandra Roncarati

**Affiliations:** 1 School of Biosciences and Veterinary Medicine, University of Camerino, Viale Circonvallazione 93-95, 62024 Matelica, MC, Italy; alberto.felici@unicam.it (A.F.); gianenrico.magi@unicam.it (G.E.M.); elisa.fiordelmondo@unicam.it (E.F.); 2 Department of Veterinary Public Health, Croatian Veterinary Institute, Savska C. 143, 10000 Zagreb, Croatia; bilandzic@veinst.hr; 3 Department of Agricultural, Environmental and Food Sciences, University of Molise, Via Francesco De Sanctis, 86100 Campobasso, CB, Italy; nicolaia@unimol.it; 4 School of Architecture and Design, University of Camerino, Viale della Rimembranza, 63100 Ascoli Piceno, AP, Italy; gerardo.doti@unicam.it

**Keywords:** sea snails, quality traits, calcium shell, by-products, sustainable harbours

## Abstract

In mid Adriatic Sea the common sea snail is the habitual snail eaten, but over the years fishermen started to capture also the long sea snail, a possible alternative for human consumption. This study aims to compare the quality traits of the edible fraction in the common and long sea snails. In this study, common and long sea snail samples were provided by fishermen in November 2018 and March 2019. Total weight (meat and shell), fractions of meat and shell (after having extracted the edible part), fatty acid, elements in meat, and calcium content in shells were determined. Meat quality traits showed high nutritional value without significant differences between the two species. The fatty acid profile showed n3/n6 ratio significantly different both considering the season of sampling (November: 4.1; March: 2.38) and the species of sea snail (common: 4.98; long: 2.86). The long species showed a higher yield in the total body and calcium content concentrations. However, the long sea snail showed 50% lower meat yield compared with the common sea snail. In conclusion, the long sea snail can be used as an alternative to the common sea snail for human consumption.

## 1. Introduction

Along the coasts of the mid-Italian Adriatic Sea of Emilia Romagna, Marche, Abruzzo and Molise, the common sea snail (*Nassarius mutabilis*) is considered the most important target species among marine snails and is particularly appreciated for traditional gastronomic dishes, known as “tiny snail” (“bomboletto” or “chiocciolino” in Italian). Gastropods are considered a valuable source of precious nutrients as protein and essential amino acids. On the Mediterranean coasts, cases of the marine snails showed high rates of n-3 long-chain polyunsaturated fatty acids, essential in preventing disorders and cardiovascular diseases [1]. Since the 1950s, the sea snail has been harvested by artisanal fishery using trawls and common cuttlefish traps. Currently, it is carried out using a basket trap called “nassino,” from the beginning of autumn to the end of spring. In relation to recent years, catch rates have decreased and an increase in the minimum landing size has been proposed [2,3]. At the same time, fishermen are recording an increase in the capture of another sea snail species, called the long sea snail or false sea snail (*Hinia reticulata*) that is considered to have no commercial value compared to the common sea snail and is thus thrown back into the sea. Both gastropods species have a similar biology, particularly living on the sandy-muddy bottoms [4].

An increase in the fishing efforts for the common sea snail (*Nassarius mutabilis*) and re-entry of the long sea snail (*Hinia reticulata*) have contributed to modifying the population stock of marine snails in different Adriatic districts [5]. Up to now, the gastronomic use of the long sea snail has not been considered because meat fraction removing the shell is more difficult than with the common sea snail. All the traditional plates are only based on the common sea snail [6]. Besides, the long one was less abundant in captures until the last few years.

Since January 2019, a ban on discards (EC Reg. 1380/2013) [7] in professional fishing has come into force and, therefore, the entire bulk of the capture operation, including non-target species, such as the specimens of the false snail, *Hinia reticulata*, must be landed (EC Reg. 1380/2013) [8]. In this situation, some fishing cooperatives have considered the possibility of employing the long sea snail species as a resource in terms of meat for human consumption [9] and shell composition in calcium content. Increasing interest in the fishery value chain and valorisation of seafood and fishery discards has promoted research on related topics as well as sustainability and a circular economy [10,11]. In addition to fishermen, other stakeholders or actors operating along the coasts are increasingly focusing on discussing the sustainable management of seafood by-products and discards. For example, after the entrance in force of the Port State Measures [12], besides the adoption of new measures to prevent illegal fishery, administrators of harbours are going to re-think the purpose of harbours in a sustainable manner. In the last few years, new models of fishery ports as infrastructure are going to be reconsidered from the environmental, economic, and social points of view [13]. At the international level, fishery harbours are moving towards a “green approach” with reduction of emissions and energy saving, and are equipped with areas dedicated to activities with a social role, such as education and training or cultural spaces, as well as processing of fish by-products [14,15]. 

From the perspective of using the two (common and long) sea snail species for purposes different to food for human consumption such as recycling by-products from the shells or meat, characterization of their quality traits in terms of meat fraction and shell was considered in this paper. Moreover, being gastropods classified as bioindicators in an aquatic environment [16], some elements were determined in the soft tissue of both species. Therefore, the edible fraction of the most appreciated common sea snail (*Nassarius mutabilis*) was evaluated in terms of proximate composition, fatty acid profile, mineral content, and heavy metals in comparison with the same traits in meat of the long sea snail (*Hinia reticulata*) species according to the catching sample. The recovery of value-added compounds, such as calcium carbonate from the shell, was also investigated in both species. The possibility of performing these processing activities in public re-designed infrastructures, such as in harbours equipped for the recovery of seafood by-products, was also proposed.

## 2. Materials and Methods

### 2.1. Samples and Sampling Areas

Sea snail samples belonging to the common (*N. mutabilis*) and long (*H. reticulata*) species were provided by fishermen catching within three miles from the seashore, along the coast of San Benedetto del Tronto at two different catching times in the first decades of November 2018 and March 2019. At each sampling, specimens of the two species of sea snail were transported on ice to the laboratory at the University of Camerino and were submitted to determination of total weight (meat + shell) and the fractions of meat and shell, after having extracted the edible part. All weight measurements were made using an electronic scale (mod. CP224S Sartorius, Gottigen, Germany). The shell yield on the total body weight was calculated (weight of shell in g/total weight of sea snail g × 100) in both groups. The samples were kept frozen and were stored at −18 °C until analysis.

### 2.2. Proximate Composition

The edible part was removed from 50 specimens/species/sampling time to determine the proximate composition and fatty acid profile. Pools of each group were homogenised and subjected to proximate analysis (moisture, protein, lipid, and ash content). The moisture percentage was determined in duplicate following the procedure of the Association of Official Analytical Chemists (AOAC) [17]. Proteins were determined using the standard Kjeldahl copper catalyst method [17]. Ash was determined using the AOAC procedure [17]. Total lipid content was measured using a modification of the chloroform:methanol procedure described by Folch et al. [18].

### 2.3. Fatty Acid Profile Determination

After determining the total lipid content of the two species of sea snails, fatty acids were converted to methyl esters following the method described by Christopherson and Glass [19]. Separation of fatty acids methyl esters was carried out on an Agilent Technologies GC/MS (6890N)/ MSD (5973inert) system (Agilent, Palo Alto, CA, USA) equipped with a db5 column (60 m × 0.25 mm) and calibrated. The operating conditions of the gas chromatograph were as follows: oven temperature was kept at 170 °C for 15 min, increased to 190 °C at a rate of 1 °C/min, then increased to 220 °C at a rate of 5 °C/min, and held at this temperature for 17 min. The temperature of the injector was 280 °C. Helium was used as the carrier gas at a constant flow of 1.0 mL/min. The identification of individual fatty acids was accomplished by comparing the observed retention times to fatty methyl esters of standard mixtures (37 FAME Mix, Supelco) and NIST MASS SPECTRAL DATABASE (NIST MS SEARCH 2.3) for mass spectrum.

### 2.4. Determination of Elements in Meat and Calcium Content in the Shell of the Two Sea Snail Species

In the two sea snail species, the concentration of essential (Se, Fe, Ca, Zn, Mg, K) and non-essential elements (Cd, Cr, Pb) in the meat fraction of a pool of samples of the two species collected in the two seasons was determined using an Agilent inductively coupled plasma with mass spectrometer (ICP-MS) system Model 7800 (Agilent, Palo Alto, CA, USA). Calibration curves were set up for quantitative determination using standard element solutions obtained by diluting the mother solution in HNO_3_ 3% + HCl 0.05% with yttrium, scandium, terbium, and bismuth as internal standards.

For shell analyses, 50 g samples of shell fraction/species, as a pool of samples in November and March, were subjected to grinding using a blender (Optimum mod. 9400 Vortex Blender) (Optimum, Bayswater, VIC, Australia) and by sieving to obtain particles less than 1 mm in size. The powdered form was then processed and the degree of CaCO_3_ was determined by treating the compound with a 1 N solution of hydrochloric acid; the excess acid was titrated with a 1 N solution of sodium hydroxide [20].

### 2.5. Statistical Analysis

Biometric parameters (total height, total weight, shell weight, meat weight) of the specimens of the two species of collected sea snails were subjected to one-way analysis of variance (ANOVA) using SPSS 25 (Version 25.0, Armonk, NY, USA) [21] and the means were considered significant at *p* < 0.01. The results concerning proximate composition, fatty acid profile, were subjected to two-way ANOVA considering the sea snail species (SP) and the season of sampling (SE) and their interaction (SP × SE) as fixed effects. Due to sample organization, element contents were subjected to one-way ANOVA considering only the snail species. In all categories of compounds, differences were considered significant at *p* < 0.01 and the means were compared using the Student–Newman–Keuls (SNK) test.

## 3. Results

In Table 1 the mean values of shell height, total weight, shell weight and mean weight were reported according to month of sampling and species. Considering the month of sampling, total weight showed significant differences with higher values in March (2.53 g) respect to November (1.38 g) whereas the meat weight was similar between the two sampling periods. In relation to the species of sea snails, the total weight showed higher values (3.86 g) in the long sea snail (*H. reticulata*) respect to the common sea snail (2.84 g). The meat weight was notably higher in the common (1.38 g) respect to the long sea snail (0.97 g). Consequently, the meat yield was double in the common compared to the long one whereas the shell yield was heavier in the long sea snail.

In Table 2 the proximate composition was reported showing contents of macronutrients very similar without notable differences independently of the season of sampling and the species of sea snail considered.

With respect to fatty acids (Table 3), considering the season of sampling, the most important category was represented by the saturated fatty acids (SFA), ranging between 45.22% and 46.24%, with palmitic acid (16:0) as the most representative fatty acid (26.29–29.08%), followed by monounsaturated fatty acids (MUFA) (22.44–23.91%), with the 18:1 (10.72–12.45%) as the most representative fatty acid. In these two categories, no significant difference was observed between the sea snails, irrespective of SE and SP. The polyunsaturated fatty acids (PUFA) n6 showed significant differences in arachidonic acid (ARA) with significantly differences between the sample of November (3.90%) and that of March (6.86%) independent of the species. The PUFA n3 exhibited significant differences in eicosapentaenoic acid (EPA) between the seasons, with the highest proportions in November (14.13%) compared to March (12.04%). Consequently, the n3/n6 ratio was significantly higher in November (4.1) compared to March (2.38).

Considering the fatty acid profile in relation to the sea snail species, independently by the season of sampling, no differences were found between the SFA in the common (47.11%) and the long one (46.43%). The two species show similar proportions of MUFA in the common (22.69%) and the long snails (20.17%). By contrast, the species type had significant effect (0.0001) on the n-6 PUFA with the highest percentage in the long species (8.13%) respect to the common (4.64%); these differences were due to the ARA which reached the prevalence in the long sea snail (6.92%) respect to the common sea snail (2.83%). The n-3 PUFA had the highest level in EPA with significantly higher percentages in the common (13.86%) respect to the long (12.89%). The n3/n6 ratio was significantly higher in the common (4.98) respect to the long sea snail (2.68).

In element concentration (Table 4), selenium content was at an average content of 24 μg/100 g in *N. mutabilis* compared to 35 μg/100 g in *H. reticulata*. The mean concentrations of other elements were similar in the common and long snails (mg/kg): Iron 3.1–4.4; calcium 32–33; zinc 1.7–1.5; magnesium 51–54; potassium 290–310. The other elements were in the ranges (μg/100 g), but significantly different with the lowest concentrations in the common sea snail: lead 26–30; cadmium 31–42; chromium 26–36.

In the gastropod shells, analyses showed that calcium was present at 283 g/kg of the long sea snail, whereas the common snail was at 265 g/kg. The purity of calcium carbonate was 96 ± 0.5% in the long snail and 91 ± 0.5% in the common one.

## 4. Discussion

In this study, the main qualitative traits of meat and the calcium shell content of the common sea snail species (*N. mutabilis*) were compared with those of the long sea snail (*H. reticulata*) whose population is increasing to the disadvantage of the most appreciated common species. To our knowledge, this paper is one of a few that deals with the quality traits of meat and the shell characterization of these gastropod species, captured in the Mediterranean Sea, which are mostly considered from the microbiological viewpoint [22]. Therefore, data were also compared with gastropods of other species and latitude distribution was investigated in a similar way to the current paper. The choice of monitoring sea snail samples at two different times (November and March) aimed to assess the quality and biometric attributes through the catching season. In the last few years, besides the revision of the minimum landing size, shortening of the fishing season (from autumn to end winter) was one of the measures proposed to safeguard the common sea snail population [23]. In our study, both in November and March, all specimens of both gastropods showed the mean shell height to be higher than that proposed as the revised minimum size (shell height 23–26 mm) [3]. Based on the results of the current study, the obligation of landing, as contemplated by the aforementioned EC Regulation, can contribute to balancing the stock population of the common species and, presumably, avoiding throwing of long sea snails back into the sea could be enough to balance the dynamics of both gastropod populations.

Analysis of the two fractions (shell, meat) of the common and long marine snails showed that the Common snail has a higher meat proportion compared to that of the Long species, which had a higher shell part, representing about 2/3 of its total weight.

The results showed that both sea snail species are a good source of protein. Among sea snails, *Rapona venosa*, collected from the Marmara Sea [24], showed high protein variation, ranging from around 65% (on dry weight) to 12.53% (on wet weight), indicating that the common and long sea snails are in a good ranking position.

With respect to the lipid content, both species exhibited low fat content in a very similar range. In the turban shell, *Turbo cornutus*, a marine snail captured along the coasts of south east Asia Japan, Korea, China, and the Philippines, lipids were determined separately in the foot and viscera and their sum appeared to be slightly higher (2.2–4.5%) than that determined in the common and long sea snails [25].

Regarding fatty acids in the soft tissue, both sea snails showed a prevalence of SFA, with palmitic acid as the most important fatty acid. This result was consistent with those in other species of gastropods from Portuguese waters, such as *Aplysia* spp. [26], but the level was lower than that reported in the hepatopancreas and meat of a *Murex* species (*Heraplex trunculus*) from the Tunisian Mediterranean coasts, collected from the fish market in a single sampling [27]. In our study, the entire edible fraction was examined in both sea snails; however, considering the mean values from those in the *Murex* species, differences were observed in fatty acid categories MUFA and total PUFA (both n3 and n6 series) as being respectively lower and higher compared to those determined in the present paper. The ARA content represented the highest difference between the common and long snail species showing higher proportions in the long compared to the common one, independent of the season. Although the samples considered in the present study were included only at the beginning and at the end of the sampling seasons and not throughout the year, the variable “season” was not significant for both gastropod species, except for ARA in the common snail where less yield was found in March. These two gastropod species, similar to most marine gastropods, are considered carnivorous with a degree of predatory activity that varies from actively seeking prey to grazing on sessile invertebrates to scavenging [22,23,24,25,26,27,28]. This feeding behaviour can be affected by the substratum and may have affected the fatty acid profile in terms of n-3 PUFA and the n3/n6 ratio in the common sea snail. *Hinia reticulata* could thus have a more generalist food spectrum including detritus and bacteria that are responsible for the higher level of ARA as determined in another gastropod (*Turbo cornutus*), collected during a year at seasonal intervals [25]. In this species of sea snail, the authors suggested that the ARA level is derived from bacteria as part of the diet, and that the low EPA level (0.48–6.12%) is probably not necessary for the survival of this mollusc [29]. Other authors [30] found that gastropods from the Mediterranean Sea (*Monodonta turbinata*, *Gibula cineraria*, *Littorina neritoides*) contained high levels of ARA but also of 18:3n-3. In the common and the long sea snails, PUFA n3, represented by EPA with a low content of 18:3n3 and DHA, were higher in proportion to that reported in a deep-vent gastropod (*Ifremeria nautilei*), defined as herbivorous, where the lack of docosaenoic acid (DHA) was dispensable [31].

The content of various elements was examined to evaluate the benefits and risks of consuming sea snails and to include them in seafood, which is an important source of high-quality proteins and essential fatty acids without adverse health effects in the full respect of food security [32,33,34]. Regarding selenium, both species of sea snails were a good source of this micronutrient, with levels comparable to those reported in fish from European aquaculture (0.02 mg/100 g and 0.06 mg/100 g fish flesh) [35] and in Japanese fish (0.12 mg/kg and 0.77 mg/kg tissue) [36].

Considering that both species were from the same sampling area, where they occupy the same trophic level, the higher concentration of non-essential elements such as lead, cadmium, and chromium, although only slightly significant, which was exhibited in the long sea snail species, can be explained by a species-specific accumulation mechanism as recognized in other aquatic organisms [37]. Different levels of essential and non-essential elements have been documented and their bioaccumulation in seafood tissues may have unwanted effects on human health. In the current paper, the sampled sea snails showed mean values below the limits of quantification imposed by the European legislation [36]. Both gastropods showed mean values lower than those reported by *N. mutabilis*, collected from the Adriatic Sea, for lead and cadmium as investigated in a previous study [38] although sampled further north (north-western Adriatic Sea) compared to our sampling area. In this previous study, toxic elements including lead and cadmium were investigated in edible echinoderms and molluscs, and showed mean values around 0.09 mg/kg (Lead) and 0.11 mg/kg (Cadmium) in the common sea snail. In our study, these elements along with zinc, iron, and magnesium were significantly lower than those reported in other gastropods (Murex spp.), for which continuous monitoring of heavy metals was recommended [39].

In relation to the calcium content in the shell, if the quality traits exhibited by the long marine snail appeared similar to those of the common species, the higher quantity of the shell in terms of yeild on the total body and calcium content, with a concentration similar to that reported in mussel shells (>280 g/kg), suggest the possibility of using the long sea snail as an alternative in food consumption. The common sea snail is requested for gastronomic uses, by contrast with the long sea snail that is considered too gummy. Another study aimed to show eventual differences in the sensory attributes of the soft tissue in these two species of sea snails, as performed for other molluscs that are appreciated just in one gourmet version, as in case of the cupped oyster [40]. 

As part of discarded materials, shells of this gastropod species can be processed to recover precious compounds such as calcium carbonate, to promote recycling of by-products. Recovery of the high calcium concentration in shells from the long sea snail is in line with the valorisation of shells from molluscs occurring in Galicia, where this practice has been promoted to employ valve mussels and to avoid incineration [41]. In bivalves, shell calcium is used as a soil amendment to increase pH in acidic soils [40,41,42]; recently, it has also been purposed as a sorbent of fluoride, a mineral associated with industrial and agricultural pollution [43]. The calcium content of the long sea snail shell could allow its inclusion in feed for aquatic species and poultry. In shrimp, growth performances were found to be enhanced by supplementation with marine snail shells, as the calcium source, especially at an inclusion level of 10% in the diet. In fact, calcium affects the moulting frequency in shrimps and tends to be absorbed into these organisms during the pre-moult period [44]. In poultry, the potential use of sea snail shells in the feeding of breeding hens could allow poultry farms to differentiate their products, thus increasing the degree of competitiveness on the market of local supply chains, which could benefit from feed supplemented with sustainable additives. In laying hens, calcium could become a supplement with high added value in feed, especially starting from the 25th week (before the deposition peak) when feed supplemented with sources of this element is necessary [45].

Management of discards derived from fishing and processing of seafood products is of growing interest at the international level [46]. Every year, the fishing industry and seafood processing sectors generate wastes mainly represented by body fractions such as head, skin, fins, viscera, mollusc valves, and crustacean exoskeletons, which are removed without any recovery attempts. Enhancement of recovery for unwanted seafood parts would reduce wastes coming from the fish sector, responding to ethical and environmental needs, but also to economic opportunities, linked with obtaining precious compounds as shown in the circular economy. According to the EC Regulation (1774/2002), by-products of fish processing plants destined for human and animal consumption are considered as Category 3 materials. A recent study [47] has quantified the amount of these waste quotas at over 60% of the biomass, focusing on the fact that their inactivity poses serious disposal and pollution problems both in developed and developing countries.

Preliminary information derived from this study suggests that in the Adriatic Sea, a “green-harbour” could be equipped as a pilot plant for treating discards of seafood processing plants or from fisheries, as the by-catch or non-target species that are obliged to be landed. This part of the harbour could have rooms equipped to perform the separation of shells for fishery by-products, in a mixture submitted to grinding, pressing to obtain a cake, followed by packaging and storage. The fishermen, organized in a sort of consortium could collect the long sea snails caught in the period in which fishing is allowed. This action could transform gastropods such as sea snails into a resource for the fishermen, penalized by the scarcity of fishery for most target species represented by the common sea snail species.

## 5. Conclusions

The proximate composition, fatty acid profile, and elemental content in the soft tissue of two marine snail species were investigated along with the calcium content in their shells. Both species of gastropods were found to be a good source of nutrients and calcium concentration. Shell composition indicated that the long sea snail can be processed to recover calcium content thus reducing waste by “closing the loop” of production in perfect agreement with the circular economy approach [10]. Operators specialized in fishing for sea snails could thus find the capture of long sea snail species as an additional economic resource by processing their shells and extracting the calcium content, indicating an interesting opportunity for commercial development.

## Figures and Tables

**Table 1:** Mean values of shell height, total weight, shell weight and meat weight of the two species of sea snails according to month of sampling (SE) and species (SP).

	Month of Sampling (SE)		Species (SP)		Error			
	November	March	*N. mutabilis*	*H. reticulata*	MSE	SE	SP	SE × SP
Shell height (mm)	25.5 ± 0.3	29.8 ± 0.5	28.6 ± 0.4	30.2 ± 0.6	1.350	2.19	0.282	n.s.
Total weight (g)	2.43 ± 0.9 B	3.45 ± 0.6 A	2.84 ± 0.6 B	3.86 ± 0.8 A	0.304	0.001	0.001	n.s.
Shell weight (g)	1.38 ± 0.8 B	2.53 ± 0.9 A	1.46 ± 0.9 B	2.89 ± 0.9 A	0.037	0.001	0.001	n.s.
Meat weight (g)	1.05 ± 0.3	0.92 ± 0.4	1.38 ± 0.3 A	0.97 ± 0.2 B	0.042	0.098	0.019	n.s.
Shell yield (%)	56.79 ± 2.4 B	73.33 ± 1.5 A	51.41 ± 1.4 B	74.87 ± 1.7 A	0.02	0.001	0.001	n.s.
Meat yield (%)	43.21 ± 1.1 A	26.67 ± 1.3 B	48.59 ± 1.6 A	25.13 ± 1.5 B	0.04	0.001	0.004	n.s.

MSE = Mean standard error; A, B = *p* < 0.01; n.s. = not significant.

**Table 2:** Proximate composition (% as it is) of the two species of sea snails according to season of sampling (SE) and species (SP).

	Month of Sampling (SE)		Species (SP)		Error			
	November	March	*N. mutabilis*	*H. reticulata*	MSE	SE	SP	SE × SP
Moisture	73.95 ± 0.9	73.72 ± 1.1	73.73 ± 1.2	72.65 ± 1.4	0.205	0.33	0.62	n.s.
Protein	21.13 ± 1.4	22.28 ± 1.3	21.24 ± 1.2	22.02 ± 1.1	0.445	0.05	0.68	n.s.
Lipids	1.95 ± 0.8	1.76 ± 0.7	1.79 ± 0.6	1.53 ± 0.9	0.877	1.4	0.16	n.s.
Ash	1.81 ± 0.4	1.83 ± 0.3	1.71 ± 0.6	1.72 ± 0.8	0.007	0.07	0.06	n.s.

MSE = Mean standard error; n.s. = not significant.

**Table 3:** Fatty acid profile (% total fatty acids) of the two species of sea snails according to season of sampling (SE) and species (SP).

	Month of Sampling (SE)		Species (SP)		Error			
	November	March	*N. mutabilis*	*H. reticulata*	MSE	SE	SP	SE × SP
SFA								
14:0	6.98	7.52	6.90	6.84	0.7230	0.2140	0.4320	n.s.
15:0	0.41	0.49	0.46	0.52	0.0734	0.0400	0.0120	n.s.
16:0	29.08	26.29	29.14	27.72	0.9611	0.930	0.0400	n.s.
17:0	1.22	1.08	1.33	1.62	0.3223	0.014	0.0140	n.s.
18:0	8.20	9.49	8.98	9.45	0.5514	0.246	0.0820	n.s.
20:0	0.34	0.35	0.30	0.28	0.1504	0.160	0.1000	n.s.
Total SFA	46.24	45.22	47.11	46.43	0.243	0.147	0.0036	n.s.
MUFA								
14:1	0.20	0.22	0.02	0.28	0.14	0.022	0.0220	n.s.
16:1	7.90	7.81	6.93	7.04	0.87	0.385	0.4950	n.s.
17:1	1.23	1.19	1.48	1.04	0.14	0.013	0.0420	n.s.
18:1	10.72	12.45	11.75	9.24	1.70	0.570	1.1600	n.s.
20:1	2.39	2.24	2.51	2.57	0.20	0.121	0.1020	n.s.
Total MUFA	22.44	23.91	22.69	20.17	1.95	0.137	0.0245	n.s.
PUFA n6								
18:2 n6	1.51	1.42	1.54	1.05	0.23	0.199	0.1730	n.s.
18:3 n6	0.25	0.25	0.27	0.16	0.18	0.047	0.0310	n.s.
20:4 n6 ARA	3.90 B	6.86 A	2.83 B	6.92 A	1.04	0.001	0.0009	n.s.
Total PUFAn6	5.66 B	8.53 A	4.64 B	8.13 A	0.23	0.0001	0.0001	n.s.
PUFA n3								
18:3 n3	2.92	2.63	3.01	3.08	0.40	0.005	0.4050	n.s.
20:5 n3 EPA	14.13 A	12.04 B	13.86 A	12.89 B	1.02	0.0003	0.0005	n.s.
22:5 n3 DPA	0.99	0.87	0.81	0.86	0.10	0.0260	0.0260	n.s.
22:6 n3 DHA	5.20	4.73	5.45	5.00	0.54	0.3850	0.1050	n.s.
Total PUFAn3	23.24 A	20.27 B	23.13	21.83	0.78	0.0001	0.2382	n.s.
Others	2.42	2.07	2.43	3.08	0.25	0.4782	0.4710	n.s.
n3/n6	4.10 A	2.38 B	4.98 A	2.68 B	0.26	0.0001	0.0001	n.s.

MSE = Mean Standard Error; A, B = *p* < 0.01; n.s. = not significant. Fatty acids are reported as numbers of C atoms (14–22) and numbers of double bonds (1–6) presents in the molecular structure, while n3 and n6 indicated the position of double bonds. SFA = Saturated Fatty Acids; MUFA = Monounsaturated fatty acids; PUFA = Polyunsaturated Fatty Acids; ARA = Arachidonic Acid; EPA = Eicosapentaenoic acid; DPA = Docosapentaenoic acid; DHA = Docosahexaenoic fatty acid.

**Table 4:** Element concentration determined in the meat of the two species of sea snails (mean value ± std. dev.).

		*N. mutabilis*	*H. reticulata*
Selenium	µg/100 g	24 ± 7	35 ± 6
Iron	mg/100 g	3.1 ± 2	4.4 ± 3
Calcium	mg/100 g	32 ± 2	33 ± 2
Zinc	mg/100 g	1.7 ± 0.3	1.5 ± 0.5
Magnesium	mg/100 g	51 ± 4	54 ± 6
Potassium	mg/100 g	290 ±13	310 ± 17
Lead	µg/100 g	26 ± 2 B	30 ± 3 A
Cadmium	µg/100 g	31 ± 5 B	42 ± 3 A
Chromium	µg/100 g	26 ± 2 B	36 ± 5 A

A, B = *p* < 0.01.
